# Assessing SARS-CoV-2 vaccine effectiveness in health workers: a cohort study conducted during the pandemic decline phase in five hospitals, affiliated to Al-Azhar University- Egypt

**DOI:** 10.1186/s12879-025-11446-9

**Published:** 2025-09-26

**Authors:** Zeinab Nabil Ahmed Said, Iman I. Salama, Samy Zaky, Sumaya H. El Shazly, Amagd A. Elzahaby, Shaaban Salah El azhary, Fathiya El-Raey, Khaled A. Eid, Areej Rushdi, Aya Ghamry, Arwa Kamhawy, Asmaa M. El-Nasser, Hazem M. El-hariri, Somaia I. Salama, Ghada A. Elshaarawy, Doaa E. Ahmed, Sherif E. Eldeeb, Rehan Saleh, Dalia M Elmosalami, Eman Elshemy, Naglaa A. Elgendy, Neamat Abdelmageed Abdelmageed, Alshaimaa M.M Eid, Shaymaa M Mohammed, Alshaymaa A. Abdel Alim, Ahmed E. Ahmed, Ahmed S. Kadah, Mohamed A. Shaheen, Abdou Mabrouk Elshafei, Ibrahim Metwally Bayoumy, Badawy W. Abobakr, Mohamed Mousa Heggazy, Ahmed M. Ghazy, Walaa M. O. Ashry, Atef W. Elrifai, Mustafa A. Haridy, Emad Abdelrazzak, Amro M. Hassan, Ahmed Qasem Mohammed, Omran Mohamed Abdelmola, Safwat Salama Sawy, Zahra Karimian, Mehrnaz Kheirandish, Kamal Fahmy, Arash Rashidian, Mahmoud Seddik

**Affiliations:** 1 Medical Microbiology and Immunology Department, Faculty of Medicine (for Girls), Al-Azhar University, Cairo, Egypt; 2 Community Medicine Research Department, Medical Research and Clinical Studies Institute, National Research Centre, P.O. 12622, Dokki, Giza, 60014618 Egypt; 3 Hepato-Gastroenterology and Infectious Diseases Department, Faculty of Medicine (for Girls), Al-Azhar University , Cairo, Egypt; 4 Hepato-Gastroenterology and Infectious Diseases Department, Faculty of Medicine, Al-Azhar University, Cairo, Egypt; 5 Hepato-Gastroenterology and Infectious Diseases Department, Faculty of Medicine, Al-Azhar University, Damitta, Egypt; 6 Hepato-Gastroenterology and Infectious Diseases Department, Faculty of Medicine, Al-Azhar University, Assiut, Egypt; 7 Clinical Pathology Department, Faculty of Medicine (for Girls), Al-Azhar University, Cairo, Egypt; 8 Dermatology and Andrology Department, Faculty of Medicine, Al-Azhar University, Cairo, Egypt; 9 Clinical Pathology Department, Faculty of Medicine, Al-Azhar University, Cairo, Egypt; 10 Medical Microbiology and Immunology Department, Faculty of Medicine (for Girls), Al-Azhar University, Damietta, Egypt; 11 Chest Department, Faculty of Medicine, Al-Azhar University, Damietta, Egypt; 12 Division of Science, Information and Dissemination, WHO Regional Office for the Eastern Mediterranean, Cairo, Egypt; 13 Heidelberg Institute of Global Health, Heidelberg University Hospital, Heidelberg, Germany; 14 Division of Communicable Diseases, WHO Regional Office for the Eastern Mediterranean, Cairo, Egypt; 15 Vice President of Postgraduate Studies and Research, Al-Azhar University, Cairo, Egypt

**Keywords:** COVID-19, Vaccine effectiveness, Binding antibodies, RT‒PCR, Health workers, Cohort study

## Abstract

**Objective:**

A cohort study was conducted with the support of the WHO, where a standardized WHO protocol was followed to assess vaccine effectiveness (VE) against symptomatic RT‒PCR confirmed SARS‒CoV-2 infection among hospital health workers (HWs) eligible for vaccination at Al-Azhar University hospitals.

**Methods:**

A WHO-supported cohort study was conducted from July 2022 through September 2023 and included 1249 HWs who were randomly selected and followed up biweekly for one year. At enrollment, nasopharyngeal (NP) and blood samples were collected from each participant and evaluated to detect SARS-CoV-2 RNA via a real-time PCR assay (QIAGEN) and for the quantitative detection of SARS-CoV-2 binding antibodies via the Roche Elecsys Anti-SARS-CoV-2 S immunoassay (Roche Diagnostics, GmbH, Germany). During follow-up, NP samples were collected from anyone who developed symptoms consistent with the WHO definition of suspected cases of SARS-CoV-2 infection.

**Results:**

At enrollment, SARS-CoV-2 RNA was detected in 119/1235 (9.6%) HWs and 89% of the participants with positive RNA were asymptomatic. COVID-19-binding antibodies were detected among 1245/1248 (99.8%) HWs, and 53.2% of them had titers > 2500 U/mL, regardless of vaccination status. During follow-up, 232 participants had COVID-19 symptoms, but only 108 provided NP samples, and 18 (16.7%) of them were positive for SARS-CoV-2 RNA. No hospitalization or mortality was recordedat enrollment or during the follow-up period. The cumulative incidence of COVID-19 infection was higher among HWs with incomplete vaccination compared to unvaccinated, fully vaccinated, or those who received booster doses (*P* = 0.025). There was no significant difference in VE among HWs who were fully vaccinated or had booster doses compared with unvaccinated HWs, with adjusted VE values of 68% (95% CI -28–92%) and 64% (95% CI -170–95%), respectively (*P* = 0.106 and 0.318 respectively). The adjusted VE increased to 89% (95% CI -33–99%) among HWs with hybrid immunity compared with those who were unvaccinated with a previous COVID-19 infection (*P* = 0.082).

**Conclusion:**

This study indicates that VE against symptomatic lab-confirmed SARS-CoV-2 infection was quite high with over 60% protection and was higher among HWs with hybrid immunity (immunity due to a combination of previous infection and vaccination) compared to unvaccinated HWs with previous COVID-19 infection. The findings also highlight the importance of completing the primary vaccination series against COVID-19. This study reveals a high rate of asymptomatic COVID-19, a lower rate of confirmed cases, who showed marked decrease in hospitalization and fatality rates. Real-world VE studies are essential to address several unresolved issues, such as the appropriate number of booster doses and the longevity of protection.

**Supplementary Information:**

The online version contains supplementary material available at 10.1186/s12879-025-11446-9.

## Introduction

 The worldwide emergence of severe acute respiratory syndrome (SARS) caused by SARS-CoV-2, which is commonly known as coronavirus disease 2019 (COVID-19), has presented a significant challenge and a global public health concern. The severity of COVID-19 spans from asymptomatic infection to critical illness, resulting in fatalities, as reported by the World Health Organization (WHO). In September 2023, there were 770,875,433 confirmed cases of SARS-CoV-2, including 6,959,316 deaths globally, 23,394,122 confirmed cases in the Eastern Mediterranean and 9,569,874 in Africa, with cumulative cases of 516,023 and 24,830 deaths in Egypt [1]. Egypt reported its first case on 14/3/2020; then experienced five waves of COVID-19 until the end of May 2022, the fifth wave starting in the first week of 2022 and lasting for 16 weeks [2, 3]. By the beginning of the fifth wave, Omicron was the dominant SARS-CoV-2 variant in Egypt [4].

Since the WHO declaration of the global pandemic of COVID-19 in March 2020 [5], several types of vaccines against COVID-19 have been rapidly developed worldwide and recent studies have confirmed safety and effectiveness of these vaccines [6, 7]. As of May 2023, a total of 13,505,262,477 vaccine doses were administered [8].Vaccination against SARS-CoV-2 is a leading strategy to change the course of the COVID-19 pandemic, as different types of COVID-19 vaccines are available worldwide. Each vaccine exhibits a different potency and duration of efficacy, as determined by the antigen design, adjuvant molecules, vaccine delivery platforms, and immunization method [9]. The CDC assesses vaccine effectiveness through multiple observational studies that utilize various methods and uses data gathered from surveillance platforms, electronic health records, and prospective studies. These studies have demonstrated that VE is influenced by several factors including host-related factors (age, chronic illness, and history of previous infection), viral factors like circulating variant(s), vaccine related factors such as vaccine type and time since vaccination, the total number of doses received, and the duration since the most recent dose [10]. A systematic literature review and meta-analysis on the effectiveness of COVID-19 vaccination against post-COVID-19 conditions (long COVID-19) among fully vaccinated individuals showed that completed COVID-19 vaccination prior to being infected resulted in a significant decrease in long COVID-19 throughout the study period. Vaccine effectiveness demonstrated an increase when booster doses were administered [11].

Egypt introduced different types of vaccines, including inactivated vaccines such as CoronaVac^®^ (Sinovac) and Covilo^®^ (Sinopharm), Beijing; adenovirus vector vaccines such as Vaxzevria^®^ (Oxford/AstraZeneca), Sputnik V^®^ (Gamaleya Institute) and Jcovden^®^ (Janssen); and mRNA vaccines such as Comirnaty^®^ (Pfizer/BioNTech) and Spikevax^®^- mRNA-1273 (Moderna) [12]. Additionally, the Egyptian Holding Company for Biological Products and Vaccines (VACSERA) started producing doses of China’s Sinovac SARS-CoV-2 vaccine [13]. Egypt started its COVID-19 vaccination rollout campaign on 24 January 2021 for medical personnel and began targeting individuals with chronic diseases and elderly individuals in March 2021. By the end of December 2023, 56% of the Egyptian population had been vaccinated with at least one dose, 41% had completed the primary vaccination series, and 15% had received at least one booster dose [14]. A large-scale national survey of 18,000 subjects was conducted by the Ministry of Health and Population from March–May 2022 in Egypt to determine COVID-19 vaccine coverage, which was low (48%) compared with the WHO 70% target [15].Thus, it is crucial to continuously evaluate and obtain updated effectiveness estimates of available vaccines against the prevalent strains of SARS-CoV-2. Real-world vaccine effectiveness (VE) studies can answer questions about effectiveness against transmission or disease outcomes by age group and risk factors, the duration of vaccine protection, relative effectiveness of different vaccines, the relative effectiveness of one dose vs. two doses or more, as well as the effectiveness of the vaccine against new viral strains. Health workers (HWs) are at increased risk of SARS-CoV-2 exposure due to frequent contact with infected patients [16]. A WHO sponsored multi-country case-control study was conducted across 62 health facilities in 16 countries (1213 cases and 1844 controls) found prolonged patient contact (< 1 m distance for > 15 min) significantly increased the likelihood of SARS-CoV-2 infection (OR 1.4; 95% CI 1.0–1.8) [17]. Notably, following the COVID-19 pandemic, there were fewer VE studies from the Eastern Mediterranean Region (EMR) due to limited research infrastructure, expertise, and financial constraints. In response, from 2021 to 2023, the Eastern Mediterranean Regional Office of the WHO (WHO-EMRO) provided technical, organizational, and financial support to enhance local research capacities and inform vaccine policies for the region. Four EMR countries– Egypt, Jordan, Iran, and Pakistan– were selected to participate, utilizing two WHO protocols: a cohort study among health workers and a test-negative design at severe acute respiratory infection surveillance sites [18]. WHO-EMRO conducted both general and country-specific workshops to build capacity in participating countries, developed tailored questionnaires uploaded to a centralized data platform (REDCap) to streamline data collection, and offered ongoing technical assistance for data quality checks and analysis to investigators. This approach enabled countries to independently analyze national VE data while facilitating pooling of data across countries and reporting regional VE estimates to inform vaccine policies. Thus, the aim of this study was to measure SARS-CoV-2 VE among HWs eligible for vaccination at Al-Azhar University hospitals against symptomatic RT‒PCR confirmed SARS-CoV-2 infection.

## Subjects and methods

### Study setting

A hospital-based prospective cohort study was carried out with support from the WHO at five university hospitals affiliated with Al-Azhar University, Egypt, focusing on HWs. Three of these hospitals are in the Cairo governorate (representing the capital), one in the Damietta governorate (representing Lower Egypt), and one in Assiut governorate (representing Upper Egypt). The WHO’s protocol, design, and methodology for assessing COVID-19 vaccine effectiveness were customized to suit the specific context and settings of the country.

### Study participants

HWs affiliated with Al-Azhar University Hospitals were randomly selected, regardless of their COVID-19 vaccination status. All categories of HWs at these hospitals were eligible for inclusion in the study, provided informed consent was obtained. Eligible participants include those who interact with patients, handle their body specimens, or manage potentially infectious waste. This encompassed physicians, nurses, emergency medical personnel, laboratory technicians, and administrative staff. To participate, HWs had to be eligible for vaccination and have no contraindications to receiving the COVID-19 vaccine. Health worker who had already received the COVID-19 vaccine through the national vaccination program outside their hospitals were also included, provided that comprehensive vaccination details were available. Those who declined to provide informed consent or were ineligible for COVID-19 vaccination due to conditions such as severe allergies or pregnancy were excluded from the study. The number of participants from each facility was estimated according to the total workforce size at each hospital: 300 participants from each hospital in Cairo, 200 from the hospital in Damietta, and 150 from the hospital in Assiut, with the total work force being 13,260 HWs in the five hospitals.

### Sampling criteria

The sampling frame for the study was derived from the updated healthcare workforce data at each university hospital, and to ensure adequate representation of specific subgroups within the workforce, stratified random sampling was conducted at each hospital. The sampling process was guided by probability proportional to size, based on job roles. A sampling interval was then calculated, and a random starting point was chosen using a table of random numbers. Out of this list, the participating HWs in each subgroup were selected based on the subgroup’s population size within each hospital.

### Participant enrollment and follow-up procedures

The study objectives were explained to HWs at the participating hospitals. Participation was voluntary, and participants could withdraw at any time without penalties, though they were asked to notify the team if they chose to do so. Enrollment took place from July to August 2022.

A face-to-face interview was carried out with each participant and a questionnaire was administered after signing an informed consent. This questionnaire included demographic, medical history, COVID- 19 vaccination history, previous COVID- 19 infections, symptoms developed and way of its diagnosis within the past year and 14 days before the interview as well as occupational and community-related behaviors. Regular ZOOM meetings were held with the research team to ensure proper study implementation at each hospital and to monitor adherence to the study protocol by the team. Participants’ self-reported vaccination status was verified through sources such as occupational health records, vaccination cards, or vaccine registries. The Ministry of Health and Population (MOH&P) provided each vaccine recipient with a card detailing their vaccine information, and vaccines were distributed to the university hospitals through a cold chain system. Participants were registered online [http://www.egcovac.mohp.gov.eg*]*, and vaccine was free of charge. Each participant who consented to take the vaccine had to fill out an application form and vaccination was postponed for 3 months for those who had a recent COVID-19 infection. Participation was encouraged through a national advertising campaign.

#### Follow-up phase

Participants in each hospital were grouped (20-25) for each investigator, who had a list of his / her assigned participants’ contact details. Participants were monitored biweekly by investigators to complete follow-up questionnaires. Regular reminders were sent before follow-up appointments. The objectives of the follow-up were to identify newly developed COVID − 19 cases among the participating HWs, track changes in their vaccination status, and to monitor changes in their potential exposure risks. Those who failed to respond within 48 h of their follow-up appointments were contacted again via an alternative way of communication. If they declined to continue, their reasons were documented. During follow up, COVID- 19 infection was identified if any participant met the following WHO COVID-19 case definition; acute onset of fever and cough OR acute onset of any three or more of the following symptoms in the previous 7 days: fever, cough, general weakness/fatigue, headache, myalgia, sore throat, coryza, dyspnea, anorexia/nausea/vomiting, diarrhea, altered mental status, anosmia, or ageusia [19]. In case of COVID-19 symptoms, the investigator examined the participant and nasopharyngeal swabs were taken within 24–48 h following the appearance of symptoms. Breakthrough infection is the term used to describe developed infections in fully vaccinated people, while non-breakthrough infection refers to infections in unvaccinated people [20] All follow-up interactions were documented using standardized written records to ensure consistency in data collection.

### Data collection procedures

The WHO provided financial and technical support for methodology, data management, report development and statistical analysis of the data [21, 22]. The data collection forms were based on the WHO protocol and relevant questionnaires [23]. Five questionnaire forms were included, each customized to fit the study requirements. The pre-enrollment and enrollment questionnaires were filled at enrollment and the follow-up questionnaire during the follow-up period. The laboratory questionnaires (serology/virology) included date, type of specimens, assigned test and results.

### Sample size calculation

The sample size was calculated based on Table 2 in the WHO protocol [24] Assuming a vaccine effectiveness of 60% and that 20% of unvaccinated HWs would be infected with SARS-CoV-2 over a period of 12 months and vaccination coverage among HWs of 90%, the required minimum sample size of 1006 participants was calculated. Accounting for an expected 20% loss to follow-up during the one-year follow-up period, 1250 participants ultimately needed to be included.

### Ethical considerations

Each participant was allocated a unique study ID number with a barcode at enrollment which was scanned at all subsequent steps to identify each respective individual’s documents and test. Name and national ID number were included in study databases. Personal identifying information was maintained only by the person responsible in each study site in accordance with regulatory agencies requirements. To ensure confidentiality, anonymization techniques were implemented by removing sensitive data including personally identifiable information, implementing safeguards against participant identification during data entry. The study was cleared by the Ethical Committee of Al- Azhar University on 5/12/2021 (AU-REC-2021-0002).

### Laboratory investigations

Nasopharyngeal swabs were transported to the main Virology Laboratory- Medical Microbiology Department, Faculty of Medicine(for girls)-Al-Azhar University and stored at −80 °C till evaluation for the presence of SARS-CoV-2 RNA via real-time RT‒PCR, whereas serum samples were transported to Clinical Pathology laboratories and stored at −20 °C, at Al-Zahraa, Al-Hussein and Bab-AlSharia Hospitals in Cairo for SARS-CoV-2-binding antibody quantitation. Follow-up NP samples were sent directly to the main virology laboratory or stored at −20 °C for a few days and then transferred to the laboratory in Cairo for analysis.

### Detection of SARS-CoV-2 binding antibodies

Total antibodies against the receptor binding domain (RBD) of the S protein were quantified in serum samples collected at enrollment via the Roche Elecsys Anti-SARS-CoV-2 S immunoassay on a Roche Cobas e 411 (Roche Diagnostics, GmbH, Germany). The limits of the blank (LoB) and limit of detection (LoD) were 0.30 U/mL and 0.40 U/mL, respectively. Test results < 0.8 U/mL were classified as nonreactive, whereas those ≥ 0.8 U/mL were classified as reactive. The upper limit of the kit was 250 U/ml, and due to a high number of positive samples that were out of range, all samples were diluted 1:10 to obtain values ≤ 2500 U/ml.

### Real-time RT‒PCR

Reverse-transcriptase polymerase chain reaction (RT-PCR) testing for SARS-CoV-2 was conducted on NP samples. This was carried out using a qualitative real-time RT-PCR assay kit (the artus^®^ SARS-CoV-2 Prep and Amp UM Kit -QIAGEN-Germany) targeting 2 viral genes (N1 and N2 of the N gene were detected through the same fluorescence channel). The two targets were not differentiated, and amplification of either or both targets led to the generation of fluorescence signal genes. The sample preparation and detection steps were integrated into a single kit with a limit of detection of 950 cp/ml. A sampling control (RNase P) and internal RNA control, together with positive and negative external controls, were included. Cycling conditions was adjusted according to the following:10 min at 50 °C for R-T reaction, followed by 2 min at 95 °C for initial activation and 40 cycles of five seconds at 95 °C and 30 s at 58 °C. Positive samples were considered when the CT was above 38 and samples were considered invalid if the channel for housekeeping gene was negative.Invalid results were obtained for a noticeable number of samples. Thus, RNA extraction (QIAamp DSP Virus spin kit) was performed for theses samples, where a larger sample volume was tested (200 µl instead of 10 µl), adequate extraction and purification of viral RNA by enzymatic lysis and mixing (protease + buffer AL with carrier RNA), and then RT‒PCR steps with the artus^®^ SARS-CoV-2 Prep and Amp UM Kit were conducted (bypassing its extraction step). The results were conclusive.

### Statistical analysis

Data entry into the REDCap platform occurred under the guidance and support of the WHO-EMRO technical team. Participant’s name, address, phone, and mobile number were excluded, as this information was kept only with the follow-up team.

Statistical analysis was carried out using SPSS for Windows, version 23. Categorical variables were presented as numbers and percentages, and continuous variables as medians and IQRs. Different statistical methods were used to assess the significance level for the differences between the study groups according to their vaccination status. Chi-square was used to detect the significant difference between categorical variables, and Fisher’s exact test was used if the expected number was below 5 in any cell. For continuous variables, one-way ANOVA was used for parametric data and the Kruskal-Wallis test for non-parametric data. Survival analysis was carried out using the Kaplan-Meier test, and a curve was generated to inspect the cumulative incidence according to vaccination status.

The incidence of SARS-CoV-2 infection was calculated as the number of events reported during the person-time at risk. For each participant, contribution of person-time at risk started from the time of enrolment, 90 days after having a SARS-CoV-2 infection confirmed by RT-PCR at or before enrolment, or 14 days after receiving 1 or 2 doses of the vaccine or 7 days from receiving booster dose, whichever was the latest. We considered 4 weeks until being at risk, for participants with a previous infection detect by only positive serology at enrolment. Person-time at risk ended on the date of onset of COVID-19 symptoms confirmed by positive RT-PCR test, the date of lost follow-up, or the date of end of the study on 15-8-2023.

### Exposure status classification and grouping for vaccine effectiveness analysis

Exposure status was classified based on the participants’ COVID-19 vaccination history at the time of being at risk. There was no change in vaccination status from enrolment to the end of follow up except for only one participant, who had received his third dose in September 2022. Participant was considered vaccinated with the first dose, 14 days after receiving the first vaccine dose (partial vaccination for all studied vaccines except mRNA-1273(Moderna) vaccine and fully vaccinated 14 days after receiving the second dose of the vaccine (for all studied vaccines except one dose only for mRNA-1273(Moderna) vaccine, and for booster after 7 days after receiving the third dose of vaccine.

Symptomatic RT-PCR confirmed SARS-CoV-2 infection was the primary outcome. Vaccine effectiveness (VE) was estimated using the unvaccinated as a reference group, unless stated otherwise. Participants were grouped according to their vaccination status, type of vaccine and time since vaccination. VE% was estimated using Cox regression proportional hazards models; hazard ratios comparing vaccinated and unvaccinated were estimated with vaccination as a time-varying exposure. VE% = 1– hazard ratio [HR]* 100, where the HR = Exp (B) in the Cox regression model. Follow-up time was from baseline to the time of symptomatic SARS-CoV-2 confirmed with positive RT-PCR or study exit. The 95% CI of VE was computed based on the 95% CI of the HR in the Cox regression.

Subgroup analyses for VE by vaccination doses (partial, fully vaccinated, and booster dose) vs. unvaccinated, different vaccine type vs. unvaccinated, then fully vaccinated vs. unvaccinated and vaccine type regarding the prior infection) were performed. For VE calculation, prior infection depends on RT-PCR positivity, either before or at enrollment, or positive serology at enrollment for unvaccinated participants. However, for prior infection, 15 participants were excluded when calculating the VEs, as 14 of them had missing RT-PCR results at enrollment (7 sample results were inconclusive / invalid and 7 were dried samples) and one missing a serology sample. These missing RT-PCR results were at random, from different hospitals.

An additional analysis was planned to stratify VE% estimates by time since vaccination. Follow-up was identified from the start of being at risk to the earliest of outcome or study exit. In the current study, the median duration from receiving the 2nd dose till the end of follow-up was 631 days (IQR: 557–730 days), and between the booster dose and the end of follow-up was 394 days (IQR: 319–491 days). Thus, we only stratified the duration into ≤ 365 days and > 365 days.

### Confounding factors and effect modifiers

Both unadjusted and adjusted estimates of VE were presented. Adjustment was made in the Cox regression model for potential confounders (age, sex, chronic comorbidities, and health facility). Hospitals were categorized in two: Cairo hospitals (Al-Zahraa, Al-Hussein, and Bab-Alsharia) and peripheral (Damiatta and Assiut). Adjustment was made in the multivariable cox regression model for all potential confounders. None of these variables were found to be significantly changing the VE% after adjustment, when multivariate Cox regression backwards Wald analysis was carried out.

### Sensitivity analysis

Sensitivity analyses were performed to estimate the robustness of the VE estimates produced in the main analysis against different assumptions (e.g., assumptions made on missing data) and/or sources of bias (e.g., the effect of unmeasured confounding factors).​

​Based on the assumptions made and on the different sources of bias identified, different sensitivity analyses were performed:​.

● Excluding participants with shorter than expected vaccination intervals between the 1st and 2nd dose and including those with < 21 days between the first and second dose of vaccine.

● Consider participants were at risk after 60 days instead of 90 days after the previous infection that detected by RT-PCR.

● Exclusion of symptomatic patients who refused to give nasopharyngeal swabs *versus* including them in the VE% estimation.

## Results

**Fig. 1:**
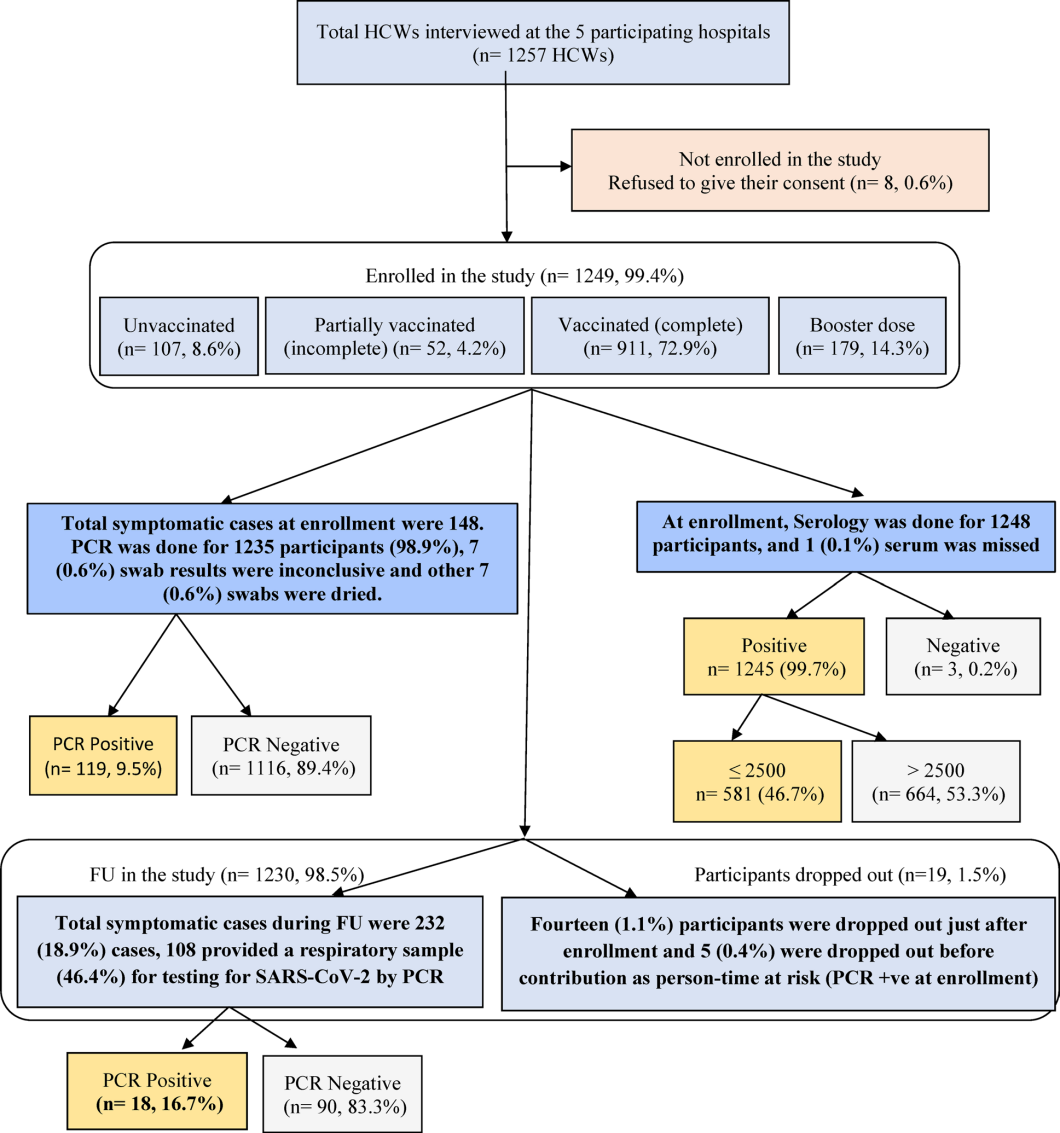
Flow chart for the study sample size, enrollment and follow- up

Out of the 13,260 HWs, 1249 were recruited into the study and agreed to be followed up biweekly for a period of one year. At enrollment, most of the included HWs, 911/1249 (72.9%), had completed the primary vaccination series (one dose of Johnson and Johnson vaccine or two doses of any other vaccine), 179 had received a booster dose (14.3%), 52 were partially vaccinated (4.2%) with one dose, and 107 (8.6%) were unvaccinated. At enrollment, COVID-19 RT‒PCR was performed for 1235 (98.8) participants, with 14 samples excluded (missed, dried or showed inconclusive results). There were 148 (11.95) symptomatic cases among the 1235 HWs at enrollment, 13 (8.8%) of them had COVID-19 RT-PCR positive results. COVID-19-binding antibody titters were measured in 1248 HWs with one missing value. At the end of the study, 1230 HWs completed the biweekly follow-up for one year, with 14 (1.1%) excluded due to dropout after enrollment and 5 (0.4%) dropped out before contribution as person-time at risk (within 90 days from the date of RT-PCR positive at enrollment). Reasons for dropout included death, retirement, change of workplace or wanted to quit from the study. Out of 1230 participants, 232 (18.9%) developed COVID-19 like symptoms, but only 108 (46.4%) agreed to provide nasopharyngeal samples. Out of them, 18 were COVID-19 RT- PCR positive (16.7%), Fig. 1.

### Baseline characteristics

**Fig. 2:**
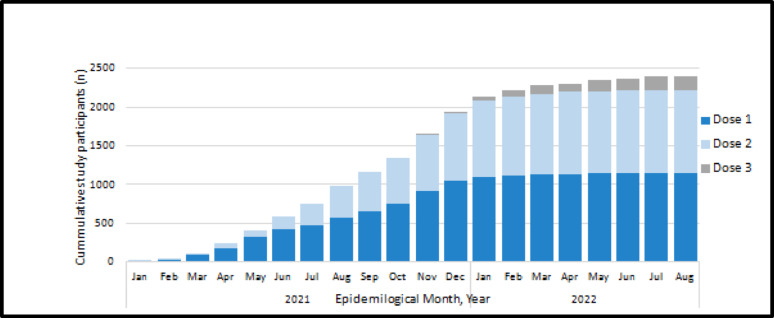
Cumulative COVID-19 vaccine coverage among study participants by epidemiological month and year

The cumulative COVID-19 vaccine coverage among the study participants is shown in Fig.2.

Their vaccination started in January 2021 and continued until August 2022. Notably, all vaccinated participants completed their primary vaccination series (full doses) before enrollment, except one, who received his / her booster dose one month after enrollment and during the follow-up period. Demographic and clinical characteristics by vaccination status at enrollment are presented in Table 1. More than half of the recruited HWs were females, 697 (55.8%), and the median age was 40.0 (IQR 30.0–49.0) years, with half of the partially vaccinated HWs were between 17 and 30 years old and 43.9% of the unvaccinated HWs were in the age group 31–40 years. Those who had completed a primary vaccination series or had a booster dose showed nearly equal age group distributions. Nearly half of the participants were nurses, 536 (42.9%). Overall, most HWs were healthy, 927(74.2%) were not reporting any chronic disease. Among those with chronic disease, hypertension and diabetes were the most common medical conditions (12.1% and 10.5%, respectively).

BBIB-CorV or WIBP-CorV, followed by ChAdOx1 nCoV-19 were the most used vaccines among the studied HWs, 481 (38.5%) and 391 (31.3%), respectively. The median and IQR between completion of the primary vaccination series and the start of follow-up was 298 days (235–388), whereas the median time between having the booster dose and the start of follow-up was 157 days (IQR: 77–201). A total of 308 (25.0%) HWs had a history of previous infection, as shown by positive RT‒PCR before/ at enrollment and/or serology positive among unvaccinated participants at enrollment, representing 164 (18.2%) of those who had completed their primary vaccination series, and 106 (100%) of the unvaccinated participants, with significant differences between groups (*P* < 0.001).

**Fig. 3:**
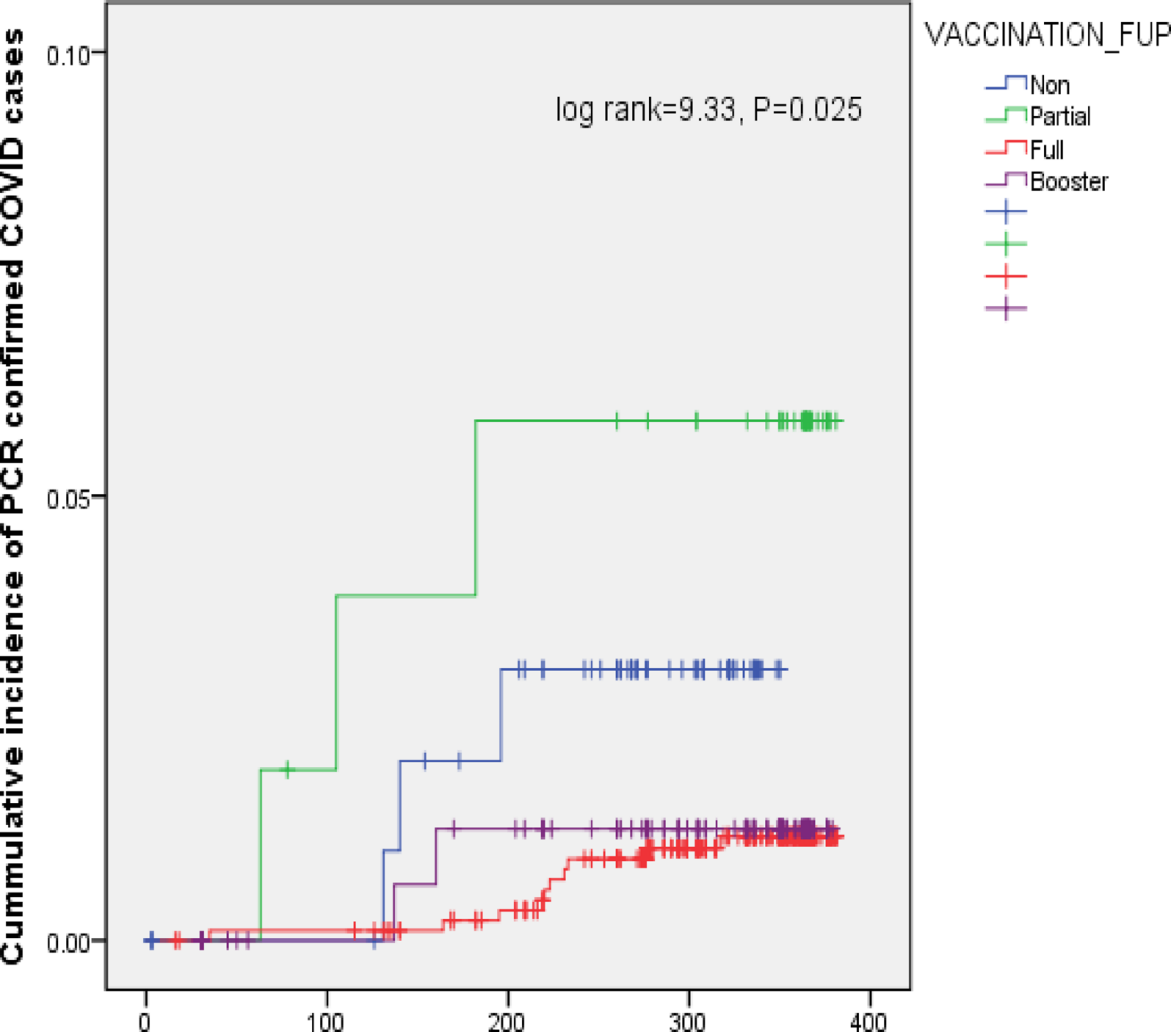
Survival Curve and Person-Time at Risk

### Follow-Up (FU)

The total follow-up period was 1148.6 person-years, and the average follow-up period was 0.94 years. Participant demographics and clinical characteristics of 1230 participants by vaccination status at the end of follow-up are presented in Table 2, where 19 participants dropped out after enrollment. Participants were followed biweekly for one year, with 232 (18.9%) HWs developing symptoms and because of individual obstacles as participant related circumstances, only 108 (46.4%) of them provided NP swabs, Fig. 1 During the follow up period, partial loss to follow up (≤ 2 visits) was recorded for 179 participants, however, they were assessed at their next visit regarding vaccination or symptoms during the missed period.

The median duration from receiving the 2nd dose until the end of follow-up was 631 days (IQR: 557–730 days), while the booster dose to the end of follow-up was 394 days (IQR: 319–491 days). The cumulative incidence of COVID-19 breakthrough infections was higher among HWs partially vaccinated than among those who were unvaccinated or those who received two or three doses of vaccine (*p* = 0.025), Fig. 3.

**Table 1:** Participant demographics and clinical characteristics by vaccination status at enrollment

Variable ©	All Participants	Unvaccinated	Partially Vaccinated *	Fully Vaccinated**	Booster Dose	*P* value
Total No. (%)	1249 (100%)	107 (8.6%)	52 (4.2%)	911 (72.9%)	179 (14.3%)	
**Age**						< 0.001
**Group(years)**						
17–30	315 (25.2%)	26 (24.3%)	26 (50.0%)	226 (24.8%)	37 (20.7%)	
31–40	329 (26.3%)	47 (43.9%)	18 (34.6%)	227 (24.9%)	37 (20.7%)	
41–50	344 (27.5%)	17 (15.9%)	5 (9.6%)	266 (29.2%)	56 (31.3%)	
> 50	261 (20.9%)	17 (15.9%)	3 (5.8%)	192 (21.1%)	49 (27.4%)	
**Median (IQR)**	40.0 (30.0–49.0)	36.0 (31.0–44.0)	30.50 (27.0–37.0)	41.0 (31.0–49.0)	43.0 (35.0–51.0)	
**Sex**						0.287
Males	552 (44.2%)	44 (41.1%)	29 (55.8%)	396 (43.5%)	83 (46.4%)	
Females	697 (55.8%)	63 (58.9%)	23 (44.2%)	515 (56.5%)	96 (53.6%)	
**Job**						
Physician	156 (12.5%)	18 (16.8%)	15 (28.8%)	101 (11.1%)	22 (12.3%)	< 0.001
Nurse	536 (42.9%)	60 (56.1%)	19 (36.5%)	370 (40.6%)	87 (48.6%)	
Technician	73 (5.8%)	4 (3.7%)	1 (1.9%)	61 (6.7%)	7 (3.9%)	
Administration	270 (21.6%)	11 (10.3%)	7 (13.5%)	224 (24.6%)	28 (15.6%)	
Workers	196 (15.7%)	12 (11.2%)	10 (19.2%)	143 (15.7%)	31 (17.3%)	
Others	18 (1.4%)	2 (1.9%)	0 (0.0%)	12 (1.3%)	4 (2.2%)	
**Chronic Illness**						
No	927 (74.2%)	83 (77.6%)	45 (86.5%)	670 (73.5%)	129 (72.1%)	0.145
Yes	322 (25.8%)	24 (22.4%)	7 (13.5%)	241 (26.5%)	50 (27.9%)	
**Vaccine Brand**						
CoronaVac	85 (6.8%)	0	6 (11.5%)	66 (7.2%)	13 (7.3%)	< 0.001
BBIB-CorV or WIBP-CorV	481 (38.5%)	0	12 (23.1%)	419 (46.0%)	50 (27.9%)	
ChAdOx1 nCoV-19	391 (31.3%)	0	26 (50.0%)	325 (35.7%)	40 (22.3%)	
Gam-COVID-Vac	22 (1.8%)	0	3 (5.8%)	7 (0.8%)	12 (6.7%)	
mRNA-1273	12 (1.0%)	0	0 (0.0%)	10 (1.1%)	2 (1.1%)	
Ad26.COV2.S	140 (11.2%)	0	5 (9.6%)	74 (8.1%)	61 (34.1%)	
BNT162b2 mRNA	11(0.9%)	0	0 (0.0%)	10 (1.1%)	1 (0.6%)	
**SARS-CoV-2 spike binding antibodies**						
**No**	3 (0.2%)	0 (1 missing)	0	3 (0.3%)	0	0.151
**Yes**	1245 (99.8%)	107 (100.0%)	52 (100.0%)	908 (99.7%)	179 (100.0%)	
**Evidence of previous infection** Ω						
No	926 (75.0%)	0 (0.0%) (1 missing)	45 (88.2%) (1 missing)	735 (81.8%) (12 missing)	146 (82.0%) (1 missing)	< 0.001
Yes	308 (25.0%)	106 (100.0%)	6 (11.8%)	164 (18.2%)	32 (18.0%)	

© The percentages reported by column

* Partially vaccinated: 1 dose of any vaccine (Johnson & Johnson is not included)

**Fully Vaccinated: one dose of Johnson & Johnson or 2 doses of any other vaccine

**€** Chronic Conditions includes Diabetes - heart disease– Hypertension - Immunodeficiency/organ transplant - lung disease– Asthma– Cancer - renal disease - Liver disease - Rheumatological disease

Ω Evidence of previous infection mean the presence of either RT-PCR positive before or at enrollment or serology positive among unvaccinated participants at enrollment

Sinovac: CoronaVac, Sinopharm: BBIB-CorV or WIBP-CorV, AstraZeneca: ChAdOx1 nCoV-19, Moderna: mRNA-1273, Janssen: Ad26.COV2. S, Pfizer: BNT162b2 mRNA

**Table 2:** Participant demographics and clinical characteristics by vaccination status at the start of Follow-Up

Variable©	All Participants*	Unvaccinated (any vaccine)	Partially vaccinated**	Fully Vaccinated***	Booster dose	*P* value
	*n* = 1230	*n* = 103 (8.4%)	*n* = 52 (4.2%)	*n* = 900 (73.2%)	*n* = 175 (14.2%)	
**Age Group (Years)**						
17–30	309 (25.1)	25 (24.3)	26 (50.0)	222 (24.7)	36 (20.6)	< 0.001
31–40	326 (26.5)	45 (43.7)	18 (34.6)	226 (25.1)	37 (21.1)	
41–50	337 (27.4)	17 (16.5)	5 (9.6)	263 (29.2)	52 (29.7)	
> 50	258 (21.0)	16(5.5)	3 (5.8)	189 (21.0)	50 (28.6)	
**Median IQR**	40.0 (30.0–49.0)	36.0 (31.0–44.0)	30.5 (27.0–37.0)	41.0 (31.0–49.0)	43.0 (34.5–51.5)	
**Sex**						
Males	545 (44.3)	44 (42.7)	29 (55.8)	390 (43.3)	82 (46.9)	0.298
Females	685 (55.7)	59 (57.3)	23 (44.2)	510 (56.7)	93 (55.1)	
**Chronic illness**						
No	914 (74.3)	80 (77.7)	45 (86.5)	662 (73.6)	127 (72.6)	0.156
Yes	316 (25.7)	23 (22.3)	7 (13.5)	238 (26.4)	48 (27.4)	
**Vaccine Brand**						
CoronaVac	85 (6.8%)	0 (0.0)	6 (11.5)	66 (7.3)	13 (7.4)	< 0.001
BBIB-CorV or WIBP-CorV	474 (38.5%)	0 (0.0)	12 (23.1)	414 (46.0)	48 (27.4)	
ChAdOx1 nCoV-19	388 (31.5%)	0 (0.0)	26 (50.0)	322 (35.8)	40 (22.9)	
Gam-COVID-Vac	1 (0.1)	0	0 (0.0%)	0 (0.0%)	1 (0.6)	
mRNA-1273	22 (1.8%)	0 (0.0)	3 (5.8)	7 (0.8)	12 (6.9)	
Ad26.COV2.S	11 (0.9%)	0 (0.0)	0 (0.0)	10 (1.1)	1 (0.6)	
BNT162b2 mRNA	136 (11.1%)	0 (0.0)	5 (9.6)	71 (7.9)	60 (34.3)	
Heterologous	10 (0.8%)	0 (0.0)	0 (0.0)	10 (1.1)	0 (0.0%)	

© The percentages reported by column

* There are 19 missing participants (14 participants were dropped out just after enrollment (death, retiring, or change of workplace)

and 5 dropped out before contribution as person-time at risk (PCR positive at enrollment)

** Partially vaccinated: 1 dose any vaccine (Johnson & Johnson is not included)

***Fully Vaccinated: 2 doses any vaccine, except for Johnson & Johnson (Janssen) only one dose considered fully vaccinated

**€** Chronic Conditions includes Diabetes - heart disease– Hypertension - Immunodeficiency/organ transplant - lung disease– Asthma– Cancer - renal disease - Liver disease - Rheumatological disease

### Laboratory results

At enrollment, SARS-CoV-2 spike-binding antibodies were detected in 1245/1248 (99.8%) of the recruited participants, Fig. 1 and Table 1. The level was > 2500 U/ml in 664/1245 (53.3%) of the recruited HWs, and three samples were negative for SARS-CoV-2 spike-binding antibodies, all of which were from HWs who had completed their primary vaccination series. All unvaccinated HWs (106, 100%) were seropositive. At enrollment, SARS-CoV-2 RNA positivity was detected among 119/1235 (9.5%) HWs. Among the 148 symptomatic HWs, only 13 (8.8%) were SARS-CoV-2 RNA positive, while most of SARS-CoV-2 RNA-positive HWs were asymptomatic (106/119–89%). Neither hospitalizations nor deaths were recorded among the recruited participants, Table 3. During the one-year follow-up period, 232 HWs developed symptoms compatible with the COVID-19 case definition, 108 (46.4%) of whom provided nasopharyngeal samples. Among these samples, 18 (16.6%) were SARS-CoV-2 RNA positive, and the peak infection rate was recorded in March 2023. They were 10 (1.1) cases among the fully vaccinated participants, 3(5.8%) among the partially vaccinated participants, 2(1.1) among those who had received a booster dose, and 3(2.9%) among unvaccinated participants, Table 3.

**Table 3:** Description of the clinical endpoints of COVID-19 RNA-Positive participants

Total Cohort	Vaccination Status at the Time of Onset				
At Enrollment	Total *n* (%)	Unvaccinated *n* (%)	Partially vaccinated* n (%)	Fully Vaccinated** n (%)	Booster *n* (%)
Total cohort with PCR swab results (14 excluded)	1235	107	51	899	178
All positive PCR	119/1235 (9.6%)	12/107 (11.2%)	4/51 (7.8%)	79/899 (8.8%)	24/178 (13.5%)
Symptomatic HWs with positive PCR	13/148 (8.8%)	1/12 (8.3%)	1/5 (20%)	8/110 (7.3%)	3/21 (14.3%)
Asymptomatic HWs with positive PCR	106/1087 (9.7%)	11/95 (11.6%)	3/46 (6.5%)	71/789 (9.0%)	21/157 (13.4%)
Symptomatic cases with positive PCR required medical care	8/13 (61.5%)	1/1 (100%)	0	4/8 (50%)	3/3 (100%)
**At End of the Follow Up (Total Cohort 1230 as 19 dropped out before being at risk)**					
Total Followed Up Participants	1230	103	52	900	175
Symptomatic HWs with Positive PCR	18/1230 (1.5%)	3/103(2.9%)	3/52 (5.8%)	10/900 (1.1%)	2/175 (1.5%)
Symptomatic HWs with Positive PCR (18) Requiring Medical Care	12 (66.7%)	0	3 (100%)	7 (70.0%)	2 (100%)

* Partially vaccinated: 1 dose of any vaccine except for Johnson and Johnson

**Fully Vaccinated: one dose of Johnson and Johnson or 2 doses of any other vaccines

### Vaccine effectiveness

#### Vaccine effectiveness per number of doses

**Fig. 4:**
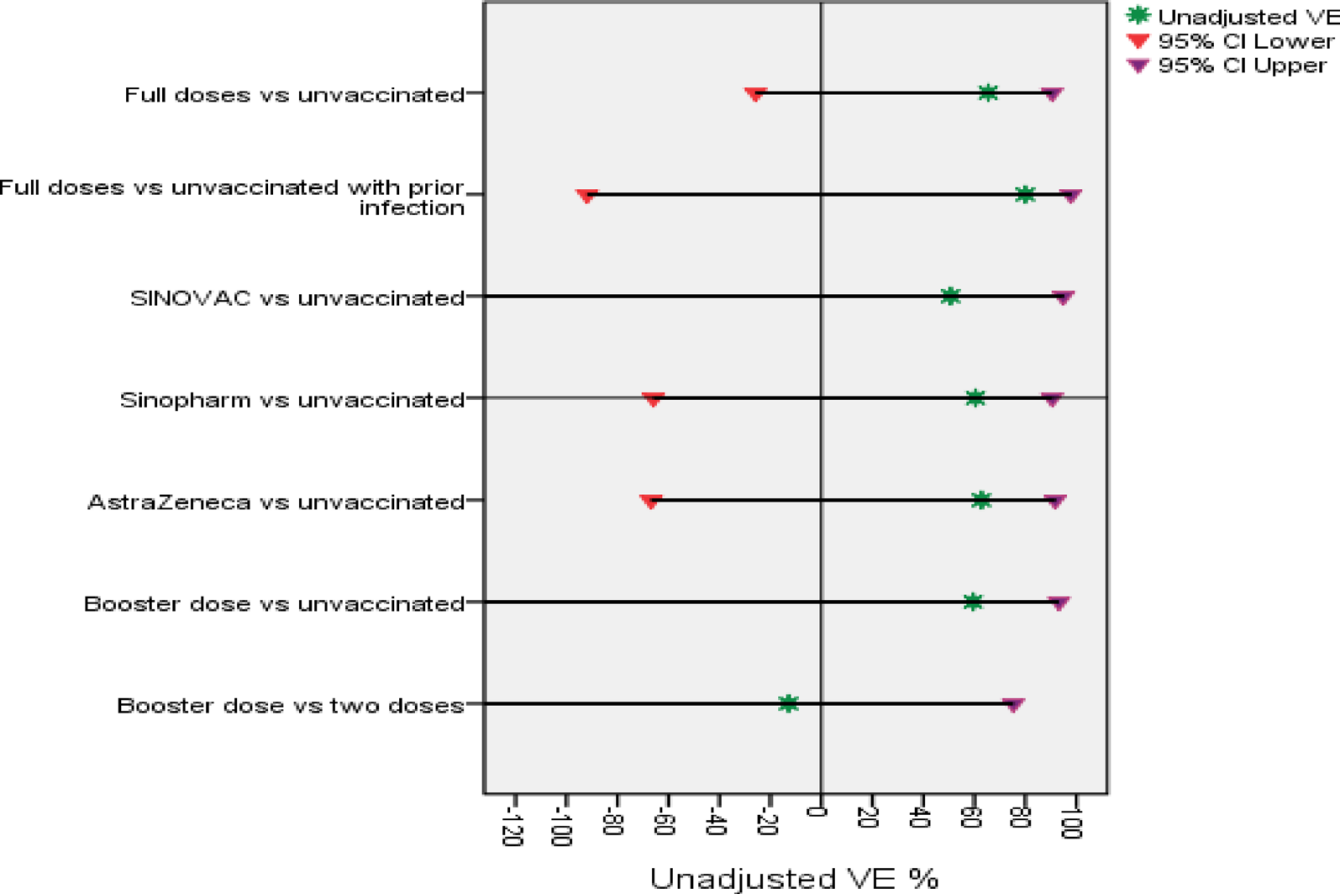
Unadjusted VE (%) against RT‒PCR-confirmed symptomatic infection

**Fig. 5:**
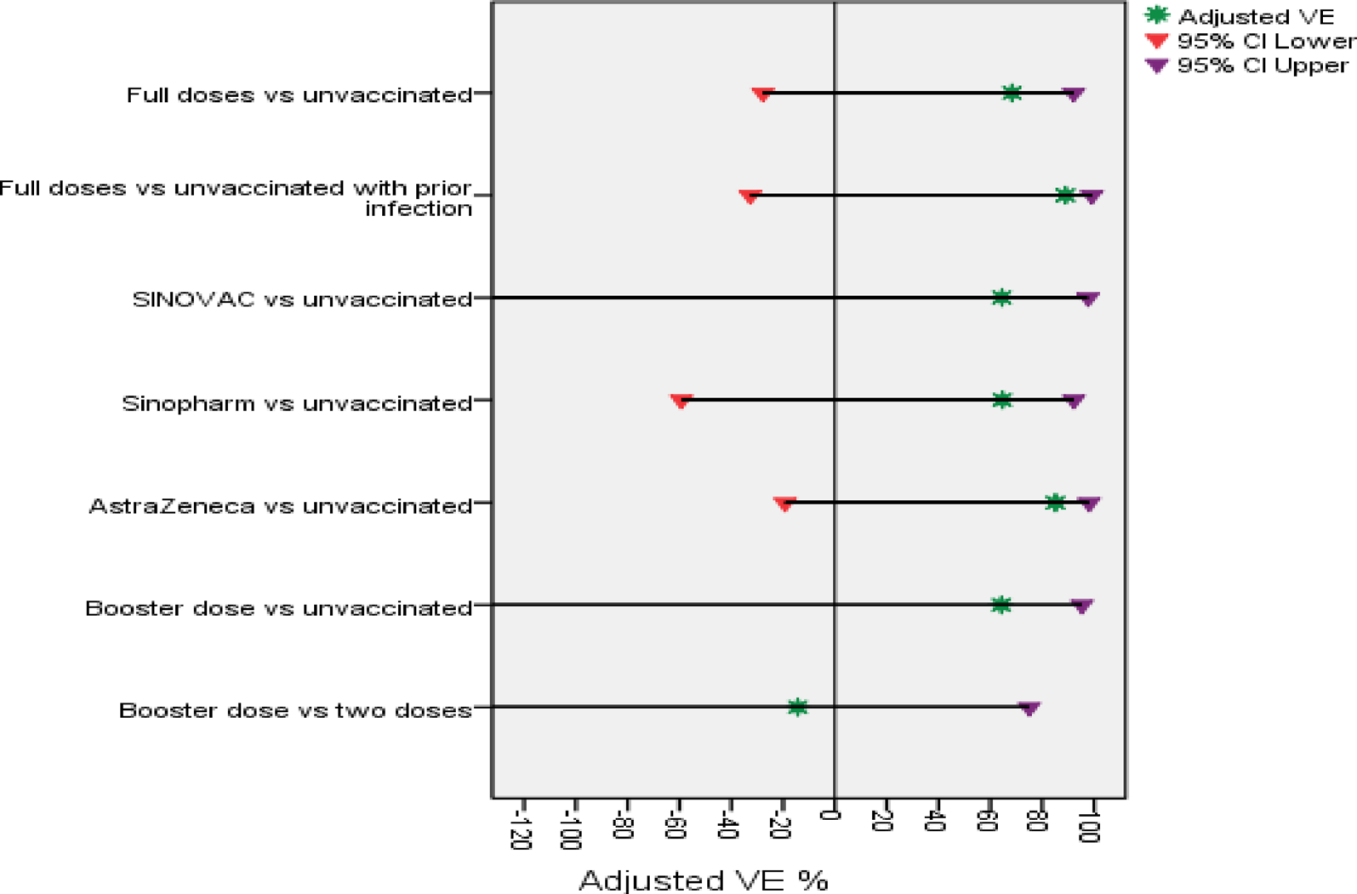
Adjusted VE (%) against RT‒PCR-confirmed symptomatic infection

The overall, VE% (including full vaccination and booster dose) was not significantly different compared with unvaccinated HWs, with unadjusted and adjusted VE values, was 64% (95%CI −26–90%). Tables 4 and Fig. 4 and 5 showed that full vaccination or booster dose VE% against symptomatic RT-PCR-confirmed COVID-19 was not significantly different compared with unvaccinated HWs, with adjusted VE% values of 68% (95%CI −28–92%) and 64% (95%CI −170–95%), respectively. Moreover, the overall VE (including partial, full vaccination and booster dose) against symptomatic RT-PCR-confirmed COVID-19 also was not significantly different compared with unvaccinated HWs, with the same unadjusted and adjusted VE value of 35% (95%CI −22–65%).

#### Vaccine effectiveness per type of vaccine

Thirteen symptomatic HWs had RT‒PCR-confirmed SARS‒CoV-2 infection, three of them being unvaccinated. One, five, and four HWs with RT-PCR confirmed COVID-19 infection were vaccinated with CoronaVac (Sinovac), BBIB-CorV or WIBP-CorV (Sinopharm) and ChAdOx1 nCoV-19 (AstraZeneca), respectively, with adjusted VE% of 65% (95%CI −59–92%) and 85% (95%CI: −19–98%) for BBIB-CorV or WIBP-CorV (Sinopharm) and ChAdOx1 nCoV-19 (AstraZeneca), respectively compared to unvaccinated group, *P* = 0.176, and *P* = 0.073 respectively. For CoronaVac (Sinovac), as there was only one symptomatic case, so VE could not be calculated, Tables 4 and Fig. 4 and 5. Because of the limited number of events, VE% was not fully calculated; however, those who had received BNT162b2 mRNA (Pfizer), mRNA-1273(Moderna), or Janssen: Ad26.COV2. S (Johnson and Johnson) did not show any breakthrough infections.

#### Vaccine effectiveness by prior infection

For prior infection, 15 participants were excluded when calculating the VEs, as 14 of them had missing RT-PCR results at enrollment. These missing PCR results were at random, from different hospitals. Among participants with prior infection (total 263), the combined effect of previous infection and vaccination (hybrid immunity) had an adjusted VE of 89% (95% CI: −33–99%), with no significant difference detected for unadjusted/adjusted VE among fully vaccination compared to the unvaccinated, *P* = 0.163 and *P* = 0.082 respectively, Tables 5, Fig. 4 and 5.

#### Vaccine effectiveness by time since vaccination

Among two-dose vaccinated participants, 10/900 developed breakthrough infection, all of whom were detected among those with a duration > 365 days from the 2nd dose with an adjusted VE of 68% (95%CI: −25–92%) compared with unvaccinated participants, *P* = 0.113. Vaccine effectiveness could not be evaluated for the duration ≤ 365 days, as none of the studied participants developed infection within this duration, Tables 6 and Fig. 4 and 5). Unadjusted and adjusted VE among participants who had received a booster dose compared with those who had received 2 vaccine doses were not significantly different (*P* > 0.05, Fig. 4 and 5).

Because of limited events, the VE in HWs receiving booster doses against RT‒PCR confirmed that COVID‒19 infection stratified by previous infection status and vaccine type could not be evaluated.

#### Sensitivity analysis of vaccine effectiveness

**Fig. 6:**
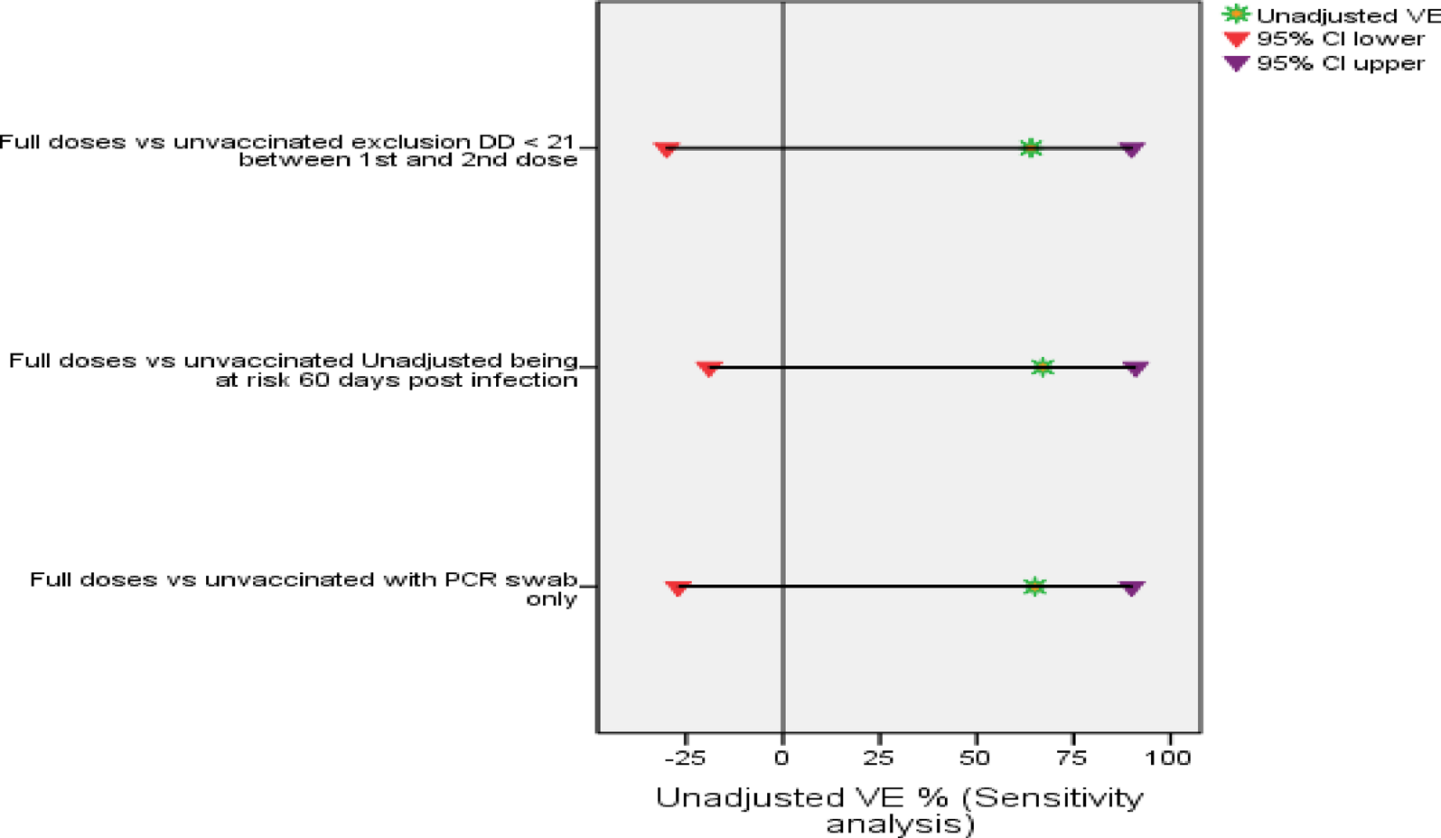
Unadjusted VE (%) against RT‒PCR-confirmed symptomatic infection (sensitivity analysis)

**Fig. 7:**
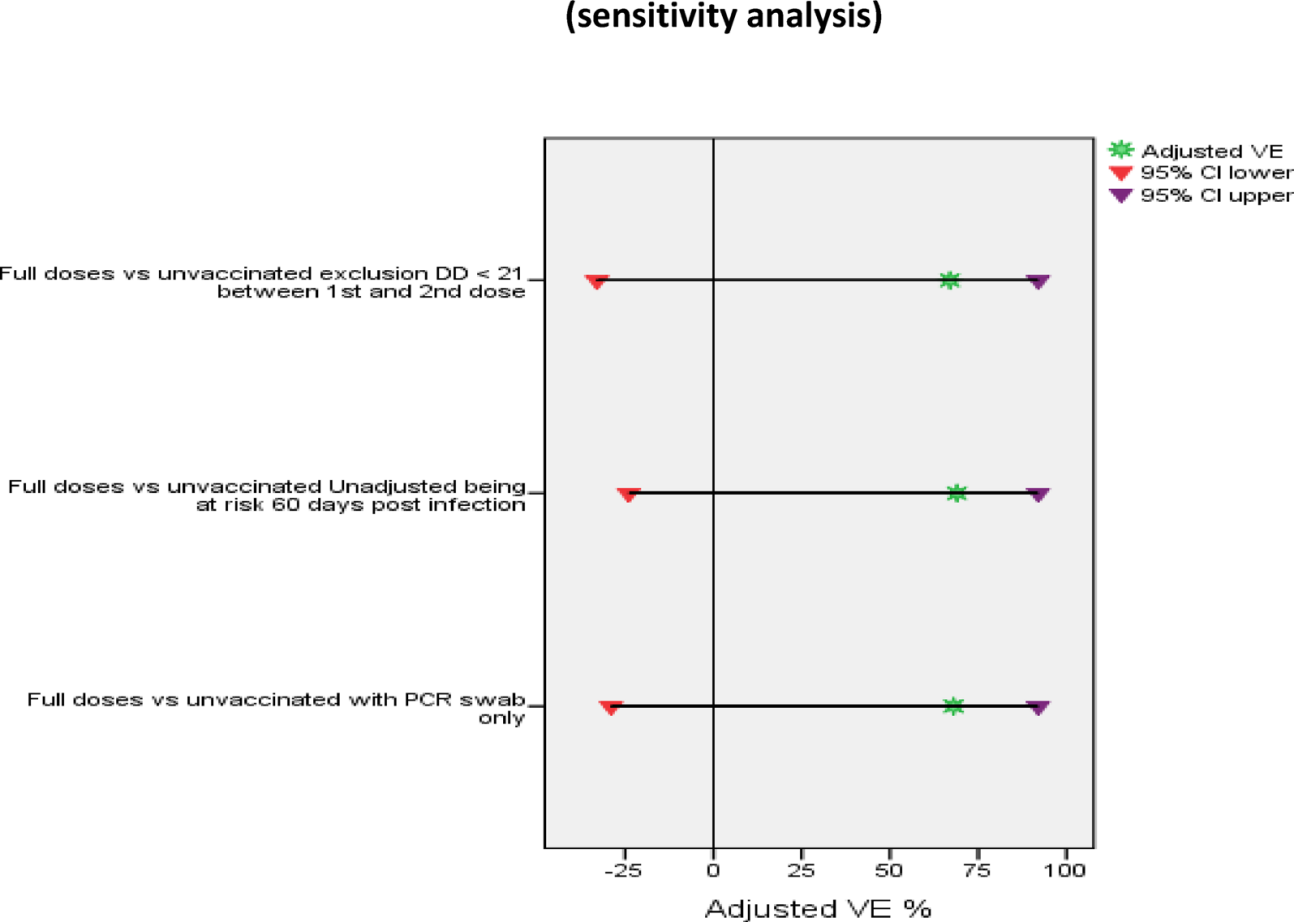
Adjusted VE (%) against RT‒PCR-confirmed symptomatic infection (sensitivity analysis)

The sensitivity analysis revealed nearly similar results for VE after the exclusion of participants with a decrease in the post-infection period at risk for 60 days instead of 90 days, with unadjusted VE values of 65% (95%CI: −27–90%), and adjusted VE 68% (95%CL: −29–92%), with P value for both = 0.111. The unadjusted VE after excluding symptomatic patients who refused to provide nasopharyngeal swabs was 67% (95%CI: −19–91%), and the adjusted VE was 69% (95%CL −24–92%) with P value for both > 0. 05. When HWs who had a duration of less than 21 days between receiving the first and second doses of the vaccine were excluded, the unadjusted VE was 64% (95%CI −30–90%) and the adjusted VE was 67% (95%CL −33–92%), with P value for both > 0.05,Fig. 6 and 7.

**Table 4:** Vaccine effectiveness against symptomatic PCR confirmed SARS-CoV-2 infection according to number of vaccine doses and vaccine type

Two Doses (All Vaccines) *	*N*	Total Person-Time (Days)	Symptomatic COVID-19 PCR-Confirmed Infections	Incidence /10,000 person-days	Unadjusted VE (95% CI)	*P* Value	Adjusted VE (95% CI)	*P* Value	
Total cohort	1230	406,382	18*	0.44					
Unvaccinated	103	29,604	3	1.01	Reference				
≥ 14days from 2nd dose	900	305,164	10	0.33	65% (−26% − 90%)	0.107	68% (−28% − 92%)	0.106	
≥ 14days from Booster dose	175	54,035	2	0.37	60% (−143% − 93%)	0.323	64% (−170% − 95%)	0.318	
By Brand of Vaccine for Total Cohort									
Total cohort*	1003	334,768	13	0.39	-	-	-	-	
Unvaccinated	103	29,604	3	1.01	Reference	-	-	-	
CoronaVac	66	22,343	1	0.45	ISD				
BBIB-CorV or WIBP-CorV	414	138,825	5	0.36	60% (−66% − 91%)	0.205	65% (−59% − 92%)	0.176	
ChAdOx1 nCoV-19	322	110,324	4	0.36	63% (−67% − 92%)	0.196	85% (−19% − 98%)	0.073	

Total cohort of the unvaccinated were 103 and fully vaccinated were 900

CI: confidence Interval VE: Vaccine Effectiveness ISD: insufficient data to calculate VE

*Three symptomatic COVID-19 PCR-confirmed infections were detected among partially vaccinated group and were excluded from the analysis of VE

Sinovac: CoronaVac, Sinopharm: BBIB-CorV or WIBP-CorV, AstraZeneca: ChAdOx1 nCoV-19

**Table 5:** Combined effect of previous infection and vaccination on VE against SARS-CoV-2 infection (Hybrid Immunity)

Two Doses (All Vaccines)	*N*	Total Person-Time (Days)	Symptomatic COVID-19 PCR-Confirmed Infections	Incidence /10,000 person-days	Unadjusted VE (95% CI)	*P* Value	Adjusted VE (95% CI)	*P* Value
Total Cohort	263	78,825	4	0.51	-	-	-	-
Unvaccinated with past infection	102	29,268	3	1.03	Reference -	-	-	-
≥ 14Days from 2nd Dose among fully vaccinated with past infection	161	49,909	1	0.20	80% (−92% − 98%)	0.163	89% (−33% − 99%)	0.082

**Table 6:** Two doses vaccine effectiveness against symptomatic COVID-19 infection, incorporating time since vaccination

Two Doses (All Vaccines)	*N*	Total person-time (days)	Symptom. COVID-19 PCR-Confirmed Infections	Incidence /10,000 person-days	Unadjusted VE (95% CI)	*P* Value	Adjusted VE (95% CI)	*P* Value
Total Cohort	1003	331,068	13	0.39				
Unvaccinated	103	29,604	3	1.01	Reference	-	-	-
14days-≤365Days from 2nd dose	7	486	0	0.00	ISD			
> 365Days from 2nd dose	893	300,978	10	0.33	64% (−27% − 90%)	0.113	68% (−25% − 92%)	0.11

CI: confidence Interval VE: Vaccine Effectiveness ISD: insufficient data to calculate VE

## Discussion

Recognizing the critical importance of leveraging local data to inform national and regional vaccine policies, the WHO-EMRO provided technical and financial support to four regional countries to conduct SARS-CoV-2 vaccine effectiveness studies [18]. Health workers (HWs) faced a significant risk of exposure to SARS-CoV-2 infection, as well as increased morbidity and mortality, while also being at a high risk of transmitting the virus to hospitalized patients, who are often more vulnerable to severe COVID-19 outcomes [25]. Thus, the current study was conducted to measure SARS-CoV-2 VE against symptomatic RT‒PCR-confirmed SARS‒CoV-2 infection among HWs eligible for vaccination at Al-Azhar University hospitals. A total of 1249 out of 13260 HWs were recruited from the workforce in the five hospitals distributed among three main governorates in Egypt with high overall vaccination coverage of 91.4% (1142 out of 1249). For the overall cohort, vaccinated participants had received their second dose long prior to enrollment (median of 294.0 days (IQR: 232–387). Several online surveys have been conducted in Egypt to study COVID-19 vaccination coverage and population perceptions, and different acceptance rates have been reported among the Egyptian population, ranging from 31.1–71.1% [3, 14, 15, 26]. An earlier questionnaire-based cross-sectional Egyptian study conducted in 2021 reported 21% vaccine coverage among HWs [27], which increased to 70.5% in a more recent similar study [28].

To study VE, unvaccinated participants were used as a reference group and VE against symptomatic COVID-19 RNA-confirmed cases was estimated. Estimates were not calculated when the total number of HWs with COVID-19 was less than five to avoid bias from limited data. Additionally, the small number of cases limited further analysis.VE estimates were often inconclusive after adjustment, likely due to the reduced statistical power. The limited number of events also resulted in significant variability in the outcomes, as evidenced by the broad confidence intervals observed for the VE estimates [25]. According to the FDA-issued guidance, the COVID-19 vaccine efficacy rate should be at least 50% to be considered successful, although the lower bound of the confidence interval can be as low as 30% [29]. In the current study, the adjusted VE among the whole study cohort was 68% (95% CI −28–92%) for those who completed their primary vaccination series. Similarly, the adjusted VE for participants who completed their primary vaccination series 68% (95% CI −28–92%) and those who received a booster dose 64% (95% CI −170–95%) showed no significant difference (*P* > 0.05). This is in line with previous studies [30]. However, it was previously shown that the booster dose remained effective for four months following vaccination and then declined substantially thereafter [30–32] Inconclusive results with wide confidence intervals were also reported in three other VE studies conducted in three different countries within the region, and this was explained by the increasing challenge of obtaining reliable VE estimates late during the COVID-19 pandemic in the Omicron dominant period [18].

Real-time RT‒PCR has been employed worldwide to detect SARS-CoV-2 due to the minimal likelihood of false positive results [33]; however, negative real-time RT‒PCR results do not exclude possible infection. Recent systematic review about the diagnostic performance of RT-PCR for detection of COVID-19 infection showed that NP swabs had better sensitivity (91.06%) compared with other clinical specimens [34]. Although the analytical performance of RT‒PCR is well accepted, pre-analytical factors cannot be excluded. At enrollment, SARS-CoV-2 RNA was detected among 119/1230 (9.6%) HWs; of those HWs, only 13 (10.9%) were symptomatic. These findings indicate that most COVID-19-positive patients are asymptomatic. However, neither hospitalization nor death were recorded among the recruited participants. This can likely be explained by occurring during the Omicron era, where the Omicron variant and its subvariants had mainly driven infections worldwide, as reported by the WHO [8]. It accounted for more than 98% of the publicly available sequences since February 2022 and constituted the genetic background from which new SARS-CoV-2 variants are likely to emerge [35]. According to the CDC, some respiratory viruses, mainly influenza and RSV, share very similar symptoms with COVID-19, which may also explain the negative RNA results for COVID-19 despite symptoms [36]. It is notable that all unvaccinated HWs were seropositive, and (77.6%) did not have RT‒PCR positive results denoting COVID-19 infection at enrollment, where the presence of seropositivity in this set of participants is indicating past infection. Of 108 (46.4%) follow-up participants, who provided a nasopharyngeal sample, 18 (16.7%) individuals were SARS-CoV-2 RNA positive, with an incidence of 0.44 cases per 10,000 person-days. Among these 18 positive cases, 15/18 (83.3) were vaccinated and 12/18 (66.7%) required medical treatment, but neither hospitalization nor death were recorded among HWs. This was in accordance with the WHO which stated that people can still develop COVID-19 after vaccination but are more likely to have mild or no symptoms [8]. Moreover, enrollment of participants occurred during the fifth wave of the COVID-19 pandemic (July and August 2022), which showed a high prevalence of Omicron variants. Compared with the early variants, Omicron variant is associated with a greater rate of transmission, a lower hospitalization rate and case fatality rate [4]. This was also confirmed in a recent Egyptian study [15].

Importantly, at enrollment, nearly all the study participants (99.8%) were seropositive for COVID-19 anti-spike-binding antibodies with levels > 2500 U/ml in most of them. Notably, only three participants (0.3%) had negative binding antibodies despite completing the vaccine doses. Moreover, all unvaccinated participants were seropositive, indicating that they had previously contracted COVID-19 infection. Anti-S and overall SARS-CoV-2-specific IgG remain detectable in approximately 90% of persons who seroconvert up to 10 months to one-year post-infection [37, 38]. Previous studies have revealed that antibody titers peak within 3–5 weeks following infection and then begin to wane in a manner that varies by individual, target antigen, antibody isotype, and assay used [37, 39]. A growing body of evidence confirms that the COVID-19 vaccine is important for preventing severe infection, hospitalization and mortality. This is in accordance with the current study, where most HWs were vaccinated and none of the HWs who experienced breakthrough infection needed hospitalization or died. Additionally, the CDC has confirmed that higher antibody titters are associated with a decreased risk of subsequent symptomatic SARS-CoV-2 infection [40].

When the binding antibody titers were evaluated in breakthrough infection, with SARS-CoV-2 after two or more doses of the vaccine, it was shown that those with breakthrough infection already had antibody titers of tens of thousands of U/mL This can be related to “hybrid immunity” after spontaneous infection following two or more doses of the vaccine [4]. Most people with COVID-19 infection develop detectable anti-SARS-CoV-2 antibodies, with seroconversion rates of 90% or higher [41, 42]. CDC reported that the immunity provided by vaccines and prior infection is high but incomplete [40]. Post-infection antibodies target the spike (S) protein, receptor binding domain (RBD) of the spike protein and nucleocapsid (N) core protein; meanwhile, vaccination induces the production of (S) and anti-RBD binding and neutralizing antibodies in the blood but not anti-N antibodies [40]. If breakthrough infections are frequent, severe, or highly transmissible, there may be a need for additional vaccine doses, adjustments to vaccine formulations, or non-pharmaceutical interventions (or a combination of these strategies) to decrease the rate of infection [20].

When the full cohort was stratified by previous infection status for full dose(s) vaccinated participants who had RT‒PCR- SARS-CoV-2 confirmed infection prior to or at enrollment (hybrid immunity), the adjusted two-dose VE vs. the unvaccinated increased to 89% CI (−33%−99%) (*P* > 0.05). Notably, the cumulative incidence of COVID-19 breakthrough infections was significantly higher among those who were partially vaccinated compared to those who were unvaccinated or those who received two or three doses of the vaccine (*p* < 0.05). This may be due to natural immunity, where all unvaccinated HWs reported previous SARS-CoV-2 infection either before or at enrollment, as detected by RT‒PCR, whereas the rates of previous SARS-CoV-2 infection among HWs with partial vaccination, those who received a full primary series or a booster dose were 11.8%, 18.2% and 18%, respectively.

Previous studies have shown that the immune response following infection continues to provide at least 50% protection against reinfection for 1–2 years following initial infection with SARS-CoV-2 [43, 44]. Additionally, in a study among HWs vaccinated 7–11 months after infection with COVID-19, the antibody titers measured 6 days after their first dose were twice as high as the antibody titers measured one month after their initial infection. Moreover, they were able to neutralize different COVID-19 variants, irrespective of vaccine type, number of doses, or pre-vaccination antibody titers [45]. A recent systematic review on measuring the magnitude and duration of hybrid immunity against severe disease and infection caused by the Omicron variant revealed that individuals with hybrid immunity had greater magnitude and durability of protection than individuals who had no history of previous infection [46]. It is worth noting that prior SARS-CoV-2 infection lowered the risk of re-infection, even without vaccination. The current findings, which show the protective effect of infection alone during the pre-Omicron period, have also been observed in other studies [25].

In the studied cohort, only seven HWs were 14–365 days from the 2nd dose; none of them developed COVID-19 symptoms during follow-up, whereas all other HWs were > 365 days from their completion of the primary vaccination series. The adjusted VE was 68% (CI −25%−92%) among HWs vaccinated vs. the unvaccinated, *P* > 0.05. A recent study indicated rapid waning of the booster dose with increased time from vaccination [47]. A model linking immunity levels and protection to VE data from England for three vaccines (Oxford/AstraZenecaAZD1222, Pfizer-BioNTech BNT162b2, Moderna mRNA-1273) and two variants (Delta, Omicron) projected gradual waning to moderate protection after 1 year [48]. The current study could not show a significant effect of a booster dose.

A recent European study showed that VE waned 12 weeks after vaccine administration and decreased even further after 24 weeks [30]. It has also been shown that the time since the last booster dose is more important than the total number of doses administered for protection against severe COVID-19 [49]. On the other hand, when VE is evaluated in relation to the vaccine brand, no significant difference was detected between each vaccine type and the unvaccinated group (*P* > 0.05). Limited infection events did not allow VE estimation among HWs in the study cohort. The VE of all the vaccines decreased over time, implying that, to reach herd immunity, booster shots are needed. VE was higher in mRNA vaccines than in the other vaccines, as no breakthrough infection was recorded among HWs receiving the BNT162b2 mRNA (Pfizer) or mRNA-1273(Moderna) vaccines, whereas the adjusted VE for ChAdOx1 nCoV-19 (AstraZeneca) was 85%, 95% CI (−19% − 98%), and that of BBIB-CorV or WIBP-CorV (Sinopharm) was 65%, 95% CI (−59% − 92%), indicating a lower VE for inactivated vaccines than for the mRNA vaccines; however, because there were few or no events, VE for CoronaVac (Sinovac) could not be calculated. This finding is in line with other studies [50]. Of note, the relative VE of the 3rd dose against RT‒PCR-confirmed COVID‒19 infection, stratified by previous infection status and vaccine type, could not be calculated due to insufficient data and limited events. Sensitivity analysis revealed similar results, with the exclusion of participants who had durations < 21 days, durations between the 1st and 2nd doses, or symptomatic patients who refused to have their nasopharyngeal swabs taken or whose disease duration decreased to 60 days instead of 90 days.

Worldwide measures were made to ensure that COVID-19 vaccines were affordable, accessible, and beneficial to the community. Due to high vaccination coverage in the study cohort, only 8% of HWs were unvaccinated, where vaccine hesitancy is still an issue of concern [4, 15]. COVID-19 vaccine refusal was multifactorial in these studies and was mainly attributed to doubts about vaccine effectiveness, a lack of trust due to rapid vaccine production, inadequate information, and a fear of vaccine side effects.

## Limitations

Some limitations were encountered, such as starting the study in the pandemic declining phase, during which too few infections occurred and that may have affected VE estimates. Additionally, VE was assessed based on laboratory-confirmed infections, using RT-PCR, that its sensitivity can be varied by several factors including timing of the sample and viral load. The low number of events underscores the need for larger sample sizes, as the small case counts often led to inconclusive VE estimates. Fewer than half of the follow-up nasopharyngeal samples were available, and the exclusion of asymptomatic individuals, who represented a significant portion of infections during the Omicron wave raises further concerns. The few events reported during follow-up limit the study’s statistical power, and most estimates were inconclusive. Also, the use of up to six different vaccine brands, combined with a limited sample size, resulted in insufficient data to calculate brand-specific VEs. Furthermore, the absence of viral genetic sequencing and the lack of anti-N antibody testing hindered the ability to distinguish between vaccine-induced and infection-induced immunity, presenting additional limitations. We also acknowledge that every study design and methodology can have certain limitations. As outlined by WHO, the cohort study approach among health workers to evaluate COVID-19 vaccine effectiveness presents few limitations, which are detailed in their guidance document. Within the manuscript, we have highlighted only the most critical limitations and have referenced the full WHO guidance for readers who may wish to explore additional potential limitations if / when deciding to adopt this methodology [51].

## Conclusions

This is the first HW cohort multicenter study conducted in Egypt to estimate VE against lab-confirmed SARS-CoV-2 infection among HWs as a frequently exposed population. Although the overall VE against symptomatic disease showed some protection, it was not significantly different from that observed in unvaccinated HWs, who showed high seroprevalence denoting previous infection. However, the protection was quite high with VEs over 60%, which is comparable with other VE studies. It is noticeable that VE was higher among HWs with hybrid immunity compared to unvaccinated HWs with previous COVID-19 infection. The point estimates and crude VE estimates support the importance of completing the primary vaccination series against COVID-19. The study also demonstrated a high rate of asymptomatic COVID-19, a lower rate of confirmed cases, who showed a marked decrease in hospitalization and fatality rates at enrollment and during follow-up period.

The study results could provide a platform for conducting and evaluating future COVID-19 VE researches, aiding policymakers in shaping national and regional vaccination strategies and understanding disease impact. This work highlights the importance of evaluating implementation challenges and feasibility, underscoring how future immunization agenda must be driven. Key unresolved issues remain such as determining the optimal number of booster doses, duration of protection and the impact of spike protein mutations in variants on vaccine efficacy.

## Supplementary information

Supplementary Material 1

## Data Availability

The data presented in the manuscript is shared with WHO regional office. Requests to access any datasets or codes/scripts used for data analysis should be directed to the corresponding author, whenever reasonable.

## Abbreviations

AB: antibody
CL: Confidence interval
COVID-19: Coronavirus disease 2019
Follow up: FU
HR: Hazard Ratio
HWs: Health workers
NP: Nasopharyngeal
RBD: Receptor Binding Domain Antibodies
RT-PCR: Reverse Transcription-Polymerase Chain Reaction
SARS-CoV2: Severe acute respiratory syndrome coronavirus 2
VE: Vaccine Effectiveness
WHO: World Health Organization

## Declarations

**Clinical trial number**.
