# Correction: Assessing SARS-CoV-2 vaccine effectiveness in health workers: a cohort study conducted during the pandemic decline phase in five hospitals, affiliated to Al-Azhar University- Egypt

**DOI:** 10.1186/s12879-025-12034-7

**Published:** 2025-11-20

**Authors:** Zeinab Nabil Ahmed Said, Iman I. Salama, Samy Zaky, Sumaya H. El Shazly, Amagd A. Elzahaby, Shaaban Salah El azhary, Fathiya El-Raey, Khaled A. Eid, Areej Rushdi, Aya Ghamry, Arwa Kamhawy, Asmaa M. El-Nasser, Hazem M. El-hariri, Somaia I. Salama, Ghada A. Elshaarawy, Doaa E. Ahmed, Sherif E. Eldeeb, Rehan Saleh, Dalia M Elmosalami, Eman Elshemy, Naglaa A. Elgendy, Neamat Abdelmageed Abdelmageed, Alshaimaa M.M Eid, Shaymaa M Mohammed, Alshaymaa A. Abdel Alim, Ahmed E. Ahmed, Ahmed S. Kadah, Mohamed A. Shaheen, Abdou Mabrouk Elshafei, Ibrahim Metwally Bayoumy, Badawy W. Abobakr, Mohamed Mousa Heggazy, Ahmed M. Ghazy, Walaa M. O. Ashry, Atef W. Elrifai, Mustafa A. Haridy, Emad Abdelrazzak, Amro M. Hassan, Ahmed Qasem Mohammed, Omran Mohamed Abdelmola, Safwat Salama Sawy, Zahra Karimian, Mehrnaz Kheirandish, Kamal Fahmy, Arash Rashidian, Mahmoud Seddik

**Affiliations:** 1 Medical Microbiology and Immunology Department, Faculty of Medicine (for Girls), Al-Azhar University, Cairo, Egypt; 2 Community Medicine Research Department, Medical Research and Clinical Studies Institute, National Research Centre, P.O. 12622, Dokki, Giza 60014618 Egypt; 3 Hepato-Gastroenterology and Infectious Diseases Department, Faculty of Medicine (for Girls), Al-Azhar University, Cairo, Egypt; 4 Hepato-Gastroenterology and Infectious Diseases Department, Faculty of Medicine, Al-Azhar University, Cairo, Egypt; 5 Hepato-Gastroenterology and Infectious Diseases Department, Faculty of Medicine, Al-Azhar University, Damitta, Egypt; 6 Hepato-Gastroenterology and Infectious Diseases Department, Faculty of Medicine, Al-Azhar University, Assiut, Egypt; 7 Clinical Pathology Department, Faculty of Medicine (for Girls), Al-Azhar University, Cairo, Egypt; 8 Dermatology and Andrology Department, Faculty of Medicine, Al-Azhar University, Cairo, Egypt; 9 Clinical Pathology Department, Faculty of Medicine, Al-Azhar University, Cairo, Egypt; 10 Medical Microbiology and Immunology Department, Faculty of Medicine (for Girls), Al-Azhar University, Damietta, Egypt; 11 Chest Department, Faculty of Medicine, Al-Azhar University, Damietta, Egypt; 12 Division of Science, Information and Dissemination, WHO Regional Office for the Eastern Mediterranean, Cairo, Egypt; 13 Heidelberg Institute of Global Health, Heidelberg University Hospital, Heidelberg, Germany; 14 Division of Communicable Diseases, WHO Regional Office for the Eastern Mediterranean, Cairo, Egypt; 15 Vice President of Postgraduate Studies and Research, Al-Azhar University, Cairo, Egypt

**Correction to: BMC Infect Dis. 2025;25:1128.**

10.1186/s12879-025-11446-9

Following publication of the original article [1], we were notified that the copyright needs to be attributed to the World Health Organization and The Authors.

The original article has been corrected.

## References

[1] Said, et al. BMC Infect Dis. 2025;25:1128. 10.1186/s12879-025-11446-9 41013275 10.1186/s12879-025-11446-9 PMC12465424

