# Supplementary figures and images for: Assessing SARS-CoV-2 vaccine effectiveness in health workers: a cohort study conducted during the pandemic decline phase in five hospitals, affiliated to Al-Azhar University- Egypt
Source: BMC Infect Dis. 2025 Sep 26;25:1128. doi: 10.1186/s12879-025-11446-9 (PMC12465424; doi:10.1186/s12879-025-11446-9)

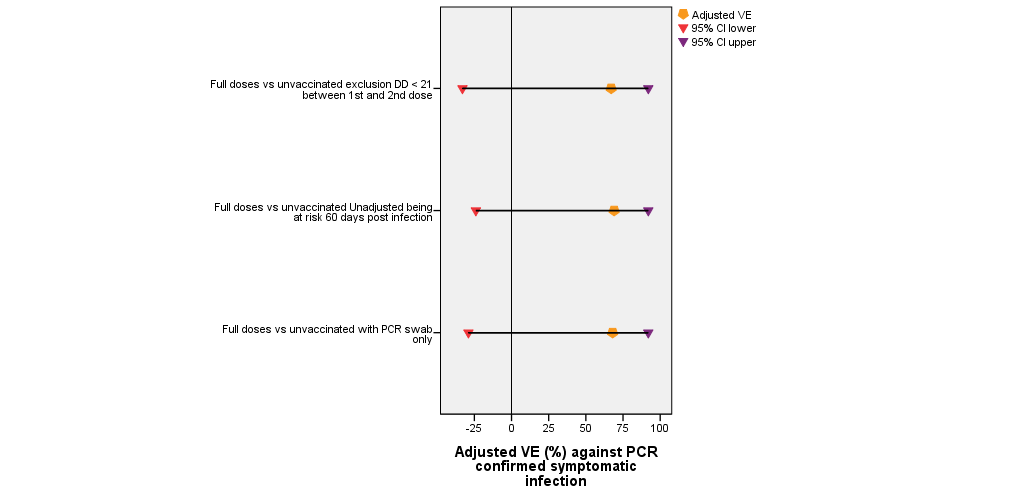

Supplement: Supplementary file 1 — Supplementary Material 1 [file 12879_2025_11446_MOESM1_ESM.zip › Fig 7.png]

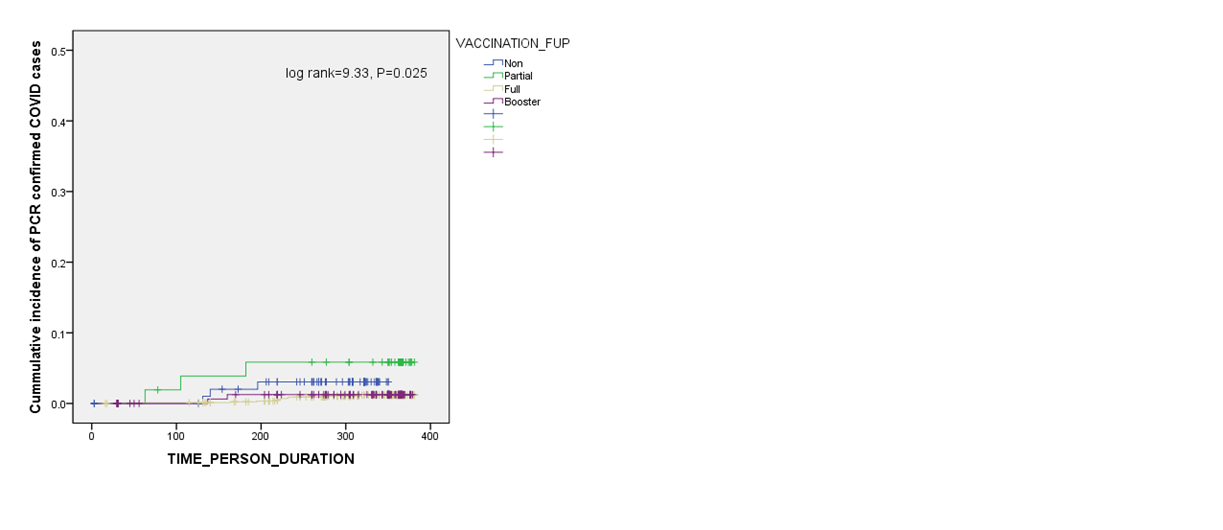

Supplement: Supplementary file 1 — Supplementary Material 1 [file 12879_2025_11446_MOESM1_ESM.zip › Fig 3.png]

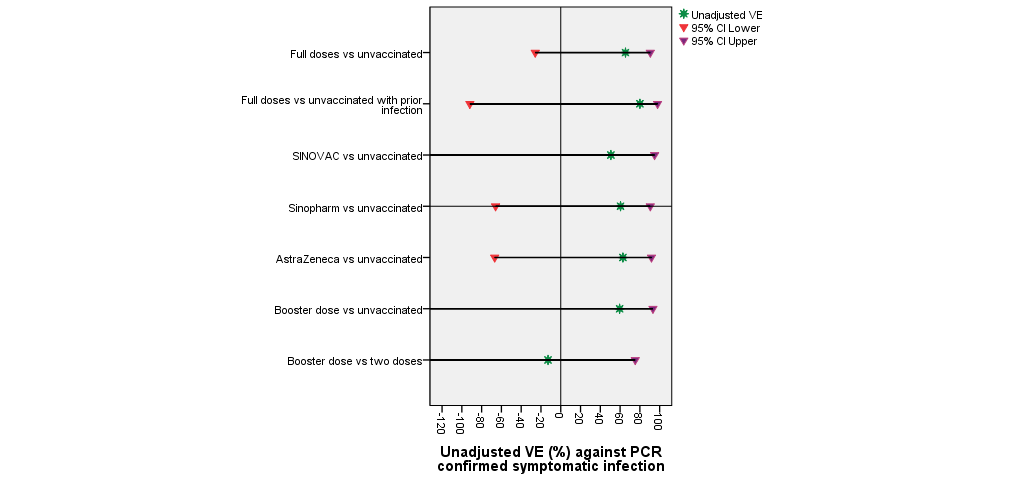

Supplement: Supplementary file 1 — Supplementary Material 1 [file 12879_2025_11446_MOESM1_ESM.zip › Fig 4.png]

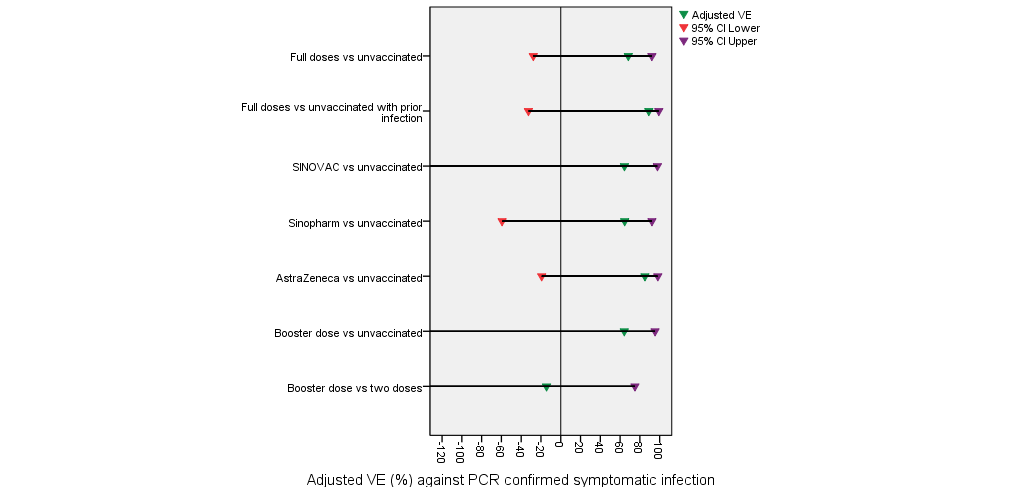

Supplement: Supplementary file 1 — Supplementary Material 1 [file 12879_2025_11446_MOESM1_ESM.zip › Fig 5.png]

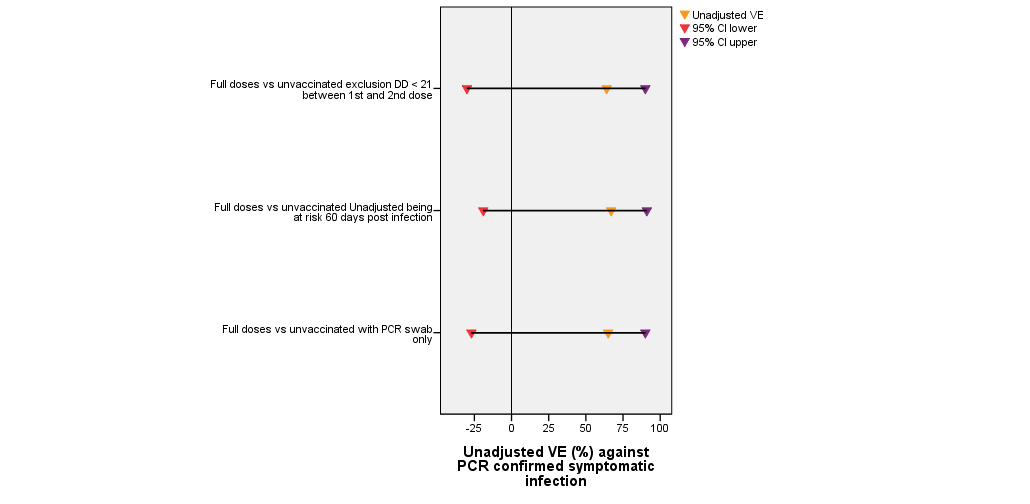

Supplement: Supplementary file 1 — Supplementary Material 1 [file 12879_2025_11446_MOESM1_ESM.zip › Fig 6.png]
